# Comparative Chondrogenic Efficacy of Dexamethasone Versus Fluocinolone Acetonide Under Varying Insulin and Glucose Concentrations in Defined Media

**DOI:** 10.3390/cells15212034

**Published:** 2026-11-07

**Authors:** Bo-An Chen, Nima Firouzi, Rodrigo A. Somoza, Harihara Baskaran

**Affiliations:** 1 Department of Chemical and Biomolecular Engineering, Center for Modular Manufacturing of Structural Tissues, Case Western Reserve University, 2102 Adelbert Road, 142 A.W. Smith Building, Cleveland, OH 44106, USA; 2 Department of Biology, Center for Modular Manufacturing of Structural Tissues, Case Western Reserve University, Cleveland, OH 44106, USA

**Keywords:** cartilage tissue engineering, human mesenchymal stem cells, glucocorticoids, chondrocyte hypertrophy, glucose metabolism, TGF-β1

## Abstract

**Highlights:**

**What are the main findings?**

- Fluocinolone acetonide (FA) promotes chondrogenesis while limiting hypertrophic differentiation: under defined TGF-β1 media conditions, FA-treated hMSC aggregates exhibit a compact morphology with robust proteoglycan (Safranin O) and collagen type II matrix deposition alongside minimal hypertrophic collagen type X staining, whereas standard dexamethasone (DXM) treatment induces collagen type X staining across multiple conditions.
- Corticosteroid selection dictates distinct metabolic and nutrient-dependent responses: DXM-treated aggregates demonstrate higher overall glucose uptake and lactate production, whereas FA-treated aggregates maintain lower baseline metabolic rates that become strongly insulin-dependent during mid- to late-stage differentiation.

**What is the implication of the main finding?**

- FA serves as a viable alternative corticosteroid for stem cell-based cartilage repair: substituting FA for DXM offers a strategy to overcome the persistent challenge of MSC hypertrophy in cartilage tissue engineering, fostering stable cartilage-like matrix synthesis with suppressed terminal maturation.
- Defined media formulation requires holistic microenvironmental optimization: designing effective chondrogenic culture protocols requires precise, synergistic tuning of corticosteroid identity together with insulin and glucose concentrations to regulate both stem cell fate and metabolic kinetics.

**Abstract:**

Chondrogenic differentiation of human mesenchymal stem cells (hMSCs) is significantly influenced by the biochemical composition of the culture microenvironment, including corticosteroid type, glucose availability, and insulin supplementation. Although dexamethasone (DXM) remains the standard glucocorticoid for chondrogenic induction, hypertrophic differentiation remains an important limitation of hMSC-based cartilage tissue engineering. Fluocinolone acetonide (FA) has shown promise in potentiating chondrogenesis with TGF-β3, but its behavior under varying metabolic conditions remains unclear. This study used a three-factor design to evaluate the effects of corticosteroid type (DXM vs. FA), glucose concentration (1 to 4.5 g/L), and insulin level (0 to 4.3 µM) on hMSC aggregate chondrogenesis over 21 days. Outcomes were assessed via biochemical assays, histology, and immunohistochemistry. FA-treated aggregates generally exhibited a smaller, more compact morphology than DXM-treated aggregates. Selected FA conditions, particularly at higher insulin concentrations, showed stronger Safranin O and collagen II staining with limited collagen X staining. DXM-treated aggregates were generally larger and more irregular and showed collagen X staining under selected low- and high-glucose conditions. DXM-treated aggregates also consumed more glucose and produced more lactate than FA-treated aggregates. These findings indicate that FA and DXM produce distinct chondrogenic and metabolic responses and suggest that FA may be used as an alternative corticosteroid for cartilage tissue engineering. The results also highlight the importance of considering both insulin and glucose concentrations when formulating defined chondrogenic media.

## 1. Introduction

Dexamethasone (DXM) is a synthetic glucocorticoid and a member of the corticosteroid hormone family [1]. Due to its potent anti-inflammatory and immunosuppressive properties, it ranks among the most widely prescribed medications globally, with broad clinical applications spanning inflammatory and autoimmune conditions [2]. However, the role of DXM in musculoskeletal tissue engineering remains paradoxical and highly context-dependent. Numerous studies have demonstrated that DXM exerts pro-anabolic and anti-catabolic effects during chondrogenesis, promoting cartilage-specific extracellular matrix synthesis while helping to suppress hypertrophic differentiation, a major challenge in cartilage tissue engineering [3,4,5]. In contrast, other studies have reported that prolonged or inappropriate DXM exposure can impair chondrogenesis by reducing chondrocyte proliferation and type II collagen synthesis, or promote osteogenic differentiation and hypertrophic maturation depending on the cell type, differentiation stage, and culture conditions [5,6]. These seemingly contradictory findings suggest that the biological effects of DXM are not universal but are strongly influenced by the cellular and biochemical microenvironment.

Unlike DXM, the potential role of alternative corticosteroids such as fluocinolone acetonide (FA) has received little attention, despite their shared glucocorticoid mechanism of action. FA has gained clinical traction in ophthalmology through sustained-release intravitreal implants, which provide effective, long-term anti-inflammatory control in chronic conditions such as diabetic macular edema and noninfectious posterior uveitis [7,8,9]. Although its therapeutic profile and local ocular side effects are investigated in retinal disease, the role of FA in chondrogenesis has barely been investigated. In a large screening study of 640 FDA-approved drugs, Hara et al. [10] identified FA as a potent catalyst for cartilage regeneration when combined with TGF-β3 (transforming growth factor-beta3). This synergistic effect is primarily mediated by FA’s ability to enhance TGF-β3-induced phosphorylation of Smad2 and Smad3 and activation of the mTORC1/AKT pathway. Both pathways have been identified as critical drivers of chondrogenic differentiation in human bone marrow-derived mesenchymal stem cells (hBMSCs). Although TGF-β3 has been reported to have a stronger chondrogenic effect on hBMSCs [11], the role of TGF-β1 in this context, particularly in combination with FA, remains relatively unexplored. Thus, the present study addresses these gaps by investigating FA-mediated chondrogenesis in a TGF-β1-based system under varying glucose and insulin conditions.

ITS (insulin–transferrin–selenium) is a standard and critical component of chemically defined chondrogenic media for human mesenchymal stem cells (hMSCs). The supplement consists of three key components: insulin, transferrin (iron transport protein), and selenium (trace element with antioxidant properties) [12]. Mueller et al. [13] found that insulin plays an essential role in chondrogenic differentiation of mesenchymal progenitor cells in vitro, showing that chondrogenic marker expression and matrix deposition improved with increasing insulin concentrations, up to an optimal level (10 µg/mL). Similarly, the effect of glucose level on chondrogenesis has been studied [14,15], which showed that increased glucose uptake has been strongly correlated with enhanced production of glycosaminoglycans (GAGs) and hydroxyproline (HYP), two key components of cartilage extracellular matrix (ECM). However, how the interplay between insulin and glucose levels shapes the chondrogenic response of hMSCs to DXM versus FA has not been systematically examined.

In this study, we aimed to evaluate the factorial effects of insulin concentration, glucose availability, and corticosteroid type (DXM vs. FA) on chondrogenic differentiation of hMSC aggregates. Multiple chondrogenic outcomes were assessed across treatment conditions. The results indicate that FA and DXM produce distinct chondrogenic and metabolic responses and suggest that FA may be used as an alternative corticosteroid for cartilage tissue engineering.

## 2. Materials and Methods

### 2.1. Materials

Bovine serum albumin, DXM and FA were purchased from Sigma-Aldrich (St. Louis, MO, USA), while selenous acid and linoleic acid (99%) were obtained from Fisher Scientific (Pittsburgh, PA, USA). TGF-β1 (10 ng/mL) and human apo-transferrin protein were acquired from R&D Systems by Biotechne (Minneapolis, MN, USA). Cell culture reagents (DMEM-LG, DMEM-HG, trypsin, L-glutamine, antibiotic/antimycotic solution, NEAA, sodium pyruvate, PBS, and nuclease-free water) were purchased from Invitrogen (Life Technologies, Carlsbad, CA, USA), along with FBS (Lot #1805387). Ascorbate-2-phosphate (A2P) was obtained from Wako Chemicals (Richmond, VA, USA), and FGF-2 from Peprotech (Rocky Hill, NJ, USA). Safranin-O, papain, cysteine, 4-(dimethylamino) benzaldehyde, H_2_SO_4_, CuSO_4_·5H_2_O, CPC, glycine, Hoechst 33258, and calf thymus DNA standard were sourced from MilliporeSigma (St. Louis, MO, USA). HCl, NaOH, sodium phosphate, Na_2_HPO_4_, NaCl, sodium acetate, EDTA, 16% formaldehyde, 30% H_2_O_2_, OCT compound, and 96-well assay plates were obtained from Fisher Scientific (Pittsburgh, PA, USA). Nitrocellulose membranes and dot-blot apparatus were purchased from Bio-Rad (Hercules, CA, USA), UV-transparent 96-well plates from Corning (Kennebunk, ME, USA), and an automatic cell counter (Countess 3) from Thermo Fisher Scientific (Waltham, MA, USA).

#### 2.1.1. Cell Culture and Chondrogenic Induction

Human bone marrow samples were obtained from healthy donors through the Hematopoietic Biorepository and Cellular Therapy Core Facility at the Case Comprehensive Cancer Center, Case Western Reserve University (Cleveland, OH, USA), under an IRB-approved protocol with informed consent, and hMSCs were isolated as previously described [16,17,18]. Cells from a single donor (a 51-year-old male) were used in this study. Cells were expanded in proliferation medium (DMEM-LG supplemented with 10% FBS and 10 ng/mL FGF-2) [19] at 1 × 10^6^ cells per T150 flask, maintained at 37 °C in a humidified atmosphere of 5% CO_2_, with medium changes every two days until 80–90% confluency was reached. Cells (passage 2) were then detached with 0.05% trypsin, counted, centrifuged, and resuspended in chondrogenic differentiation medium containing TGF-β1 and other components as previously described [20] except DXM, FA and ITS+. The cell suspension was then seeded onto conical-bottom 96-well plates at 2.5 × 10^5^ cells/well, and centrifuged to form pellets. Following two days of initial aggregation, medium was replaced with treatment-specific chondrogenic medium every two days for 21 days.

#### 2.1.2. Experimental Design

Five ITS+ solutions were prepared in PBS, each containing a designated concentration of human recombinant insulin (0, 0.3, 1, 2.5, or 4.3 µM) and consistent concentrations of human transferrin (0.625 mg/mL), selenous acid (625 ng/mL), BSA (125 mg/mL), and linoleic acid (0.535 mg/mL). This range includes the concentration used in standard ITS supplementation (about 1 µM) and extends above it. The higher levels (2.5 and 4.3 µM) remain within the range previously examined in human MSC chondrogenic cultures (0 to 50 µg/mL) [13]. Glucose concentrations of 1, 2, 3, and 4.5 g/L were prepared by mixing DMEM containing 4.5 g/L glucose levels (DMEM-HG) and DMEM containing no glucose (DMEM-NG). DXM or FA was added to a final concentration of 100 nM in chondrogenic media. A three-factor experimental design was employed to evaluate the effects of glucose concentration, insulin levels, and glucocorticoid type (DXM vs. FA) on chondrogenic differentiation, as outlined in Table 1 and Table 2. In this experiment, nine independent biological replicates were used for each treatment. From these, three aggregates were allocated for histology and IHC staining and four for biochemical measurements. However, for some treatments, one to three samples were lost during processing because the aggregates were very small.

#### 2.1.3. Imaging and Image Analysis

Images of all harvested aggregates on Day 21 were obtained using a stereo microscope (Leica Microscope, Wetzlar, Germany). ImageJ (https://github.com/imagej/ImageJ, accessed on 20 February 2026) was used to analyze the images to obtain projected diameter and circularity [21].

#### 2.1.4. DNA/GAG/HYP Assay

Aggregates were harvested on Day 21 and analyzed for biochemical content. Quantitative analyses of GAG and DNA content were performed as previously described [16,18,22]. HYP content was quantified using a previously established assay [23]. GAG and HYP content for both treatments were averaged for four aggregates (n = 4) for each condition, and mean values were illustrated as heat maps. However, DNA, GAG/DNA, and HYP/DNA values are presented in Supplementary Materials (Figures S7–S9).

#### 2.1.5. Glucose/Lactate Assay

Glucose levels in the collected media were measured using a CareLiving blood glucose meter (Model BGM101; CareLiving Diagnostics Inc., Hangzhou, China). Glucose uptake (µmol) was determined by mass balance over each two-day interval between medium changes. Cumulative glucose uptake (µmol) at each time point was calculated by summing the uptake over all preceding intervals. Glucose uptake rate (µmol/day) was calculated by dividing the uptake during each interval by its duration. Lactate concentrations were measured using a previously reported enzymatic assay [24]. Lactate secretion (µmol) was calculated from the measured lactate concentration and medium volume. Cumulative lactate secretion (µmol) and lactate secretion rate (µmol/day) were calculated in the same manner as for glucose uptake. For longitudinal glucose and lactate measurements, the same aggregate was followed throughout the culture period. For developmental-stage analyses, interval-specific glucose uptake and lactate secretion rates were averaged within each aggregate over early (Days 0–4), mid (Days 6–12), and late (Days 12–21) culture periods. The Day 6 measurement was excluded from these analyses because lactate measurements were unrecoverable for a subset of FA samples. Values are reported as mean ± standard deviation (SD) for three biological replicates (n = 3).

#### 2.1.6. Histological Staining

Paraffin-embedded aggregates fixed in 4% formaldehyde were sectioned at 5 µm and stained with Safranin-O following standard protocols [25]. The stained sections were imaged using an inverted microscope (BZX800, Keyence, Osaka, Japan). The Safranin-O-stained regions from three sections over 2–3 aggregates for a given condition were quantified using ImageJ (version 1.53k) by color thresholding and expressed as a percentage of the total aggregate area.

#### 2.1.7. Immunohistochemistry (IHC)

IHC was performed on paraffin sections from three biological replicates (n = 3) per group to detect collagen II and collagen X. After deparaffinization and rehydration, antigen retrieval was carried out with proteinase K (10 µg/mL; IHC Select, Cat. No. 21627, MilliporeSigma, St. Louis, MO, USA) for 25 min at 37 °C, and endogenous peroxidase was quenched with 3% hydrogen peroxide for 30 min. Sections were blocked with 2.5% normal horse serum for 20 min and incubated overnight at 4 °C with mouse monoclonal antibodies against collagen II (clone 5B2.2, Cat. No. 605–640, Invitrogen, Carlsbad, CA, USA) or collagen X (clone X53, Cat. No. 14-9771-82, Invitrogen), each diluted 1:100. Detection used the ImmPRESS HRP Horse Anti-Mouse IgG Polymer Kit (Cat. No. MP-7402, Vector Laboratories, Newark, CA, USA) for 1 h, followed by ImmPACT NovaRED HRP Substrate (Cat. No. SK-4805, Vector Laboratories) for 10–30 min. Sections were then rinsed, cover slipped, and stored at 4 °C. The stained sections were imaged using an inverted microscope (BZX800, Keyence). The immunostained regions from three sections over 2–3 aggregates for a given condition were quantified using ImageJ (version 1.53k; National Institutes of Health, Bethesda, MD, USA) by color thresholding and expressed as a percentage of the total aggregate area.

#### 2.1.8. Statistical Analysis

Longitudinal glucose uptake and lactate secretion rates were analyzed using generalized estimating equations (GEE) with an autoregressive [AR(1)] correlation structure to account for repeated measurements from the same aggregate [26,27]. Corticosteroid (DXM or FA), insulin concentration, initial glucose concentration, and culture time were included as categorical factors. The model evaluated main effects and interactions among corticosteroid, insulin, and glucose, as well as the interactions of each factor with time. Measurements identified as unrecoverable or as technical assay failures were treated as missing and were not used. For developmental-stage analyses, interval-specific rates were averaged within each aggregate over early (Days 0–4), mid (Days 6–12), and late (Days 12–21) culture periods. The Day 6 measurement was excluded from these analyses because lactate measurements were unrecoverable for a subset of FA samples.

## 3. Results

### 3.1. Morphology

**Figure 1:**
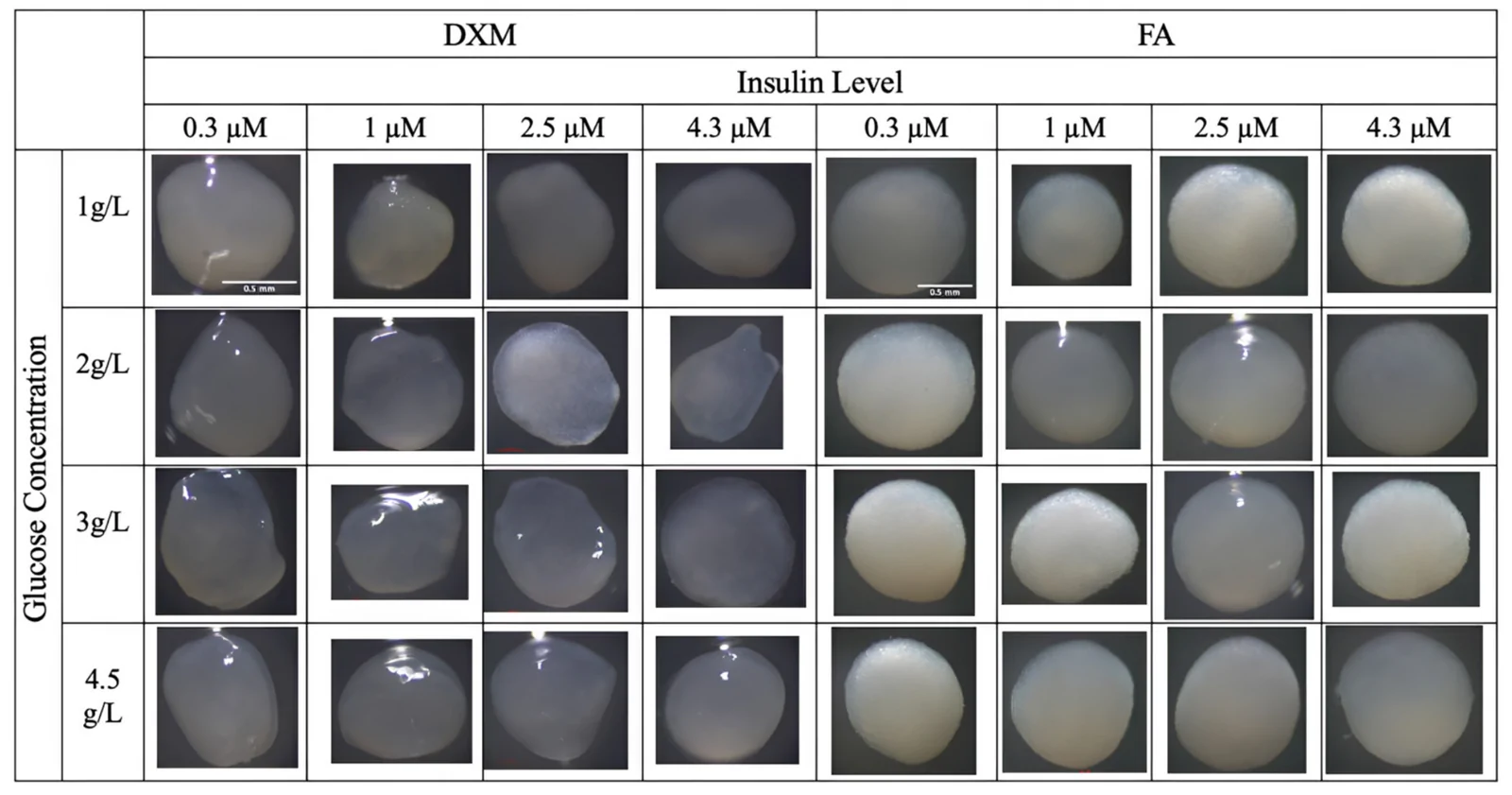
Gross morphology of chondrogenic aggregates at Day 21 under stereomicroscopy across varying corticosteroid type (DXM vs. FA), glucose concentration (1, 2, 3, and 4.5 g/L), and insulin level (0.3, 1, 2.5, and 4.3 µM). Scale bar = 0.5 mm shown only for a sample of aggregates (n = 8–9).

FA-treated aggregates were generally smaller, more circular, and more compact than DXM-treated aggregates (Figure 1). The morphology data (diameter and circularity of the aggregates) are shown in Supplementary Materials Tables S1 and S2. Most FA-treated aggregates had circularity values above 0.90, whereas several DXM-treated aggregates had values below 0.70. DXM-treated aggregates also showed greater variation in shape and were generally more translucent than FA-treated aggregates. In the absence of insulin, aggregates exhibited less organized morphology and smaller diameters (see Supplementary Material, Figure S1).

### 3.2. Biochemical Content

**Figure 2:**
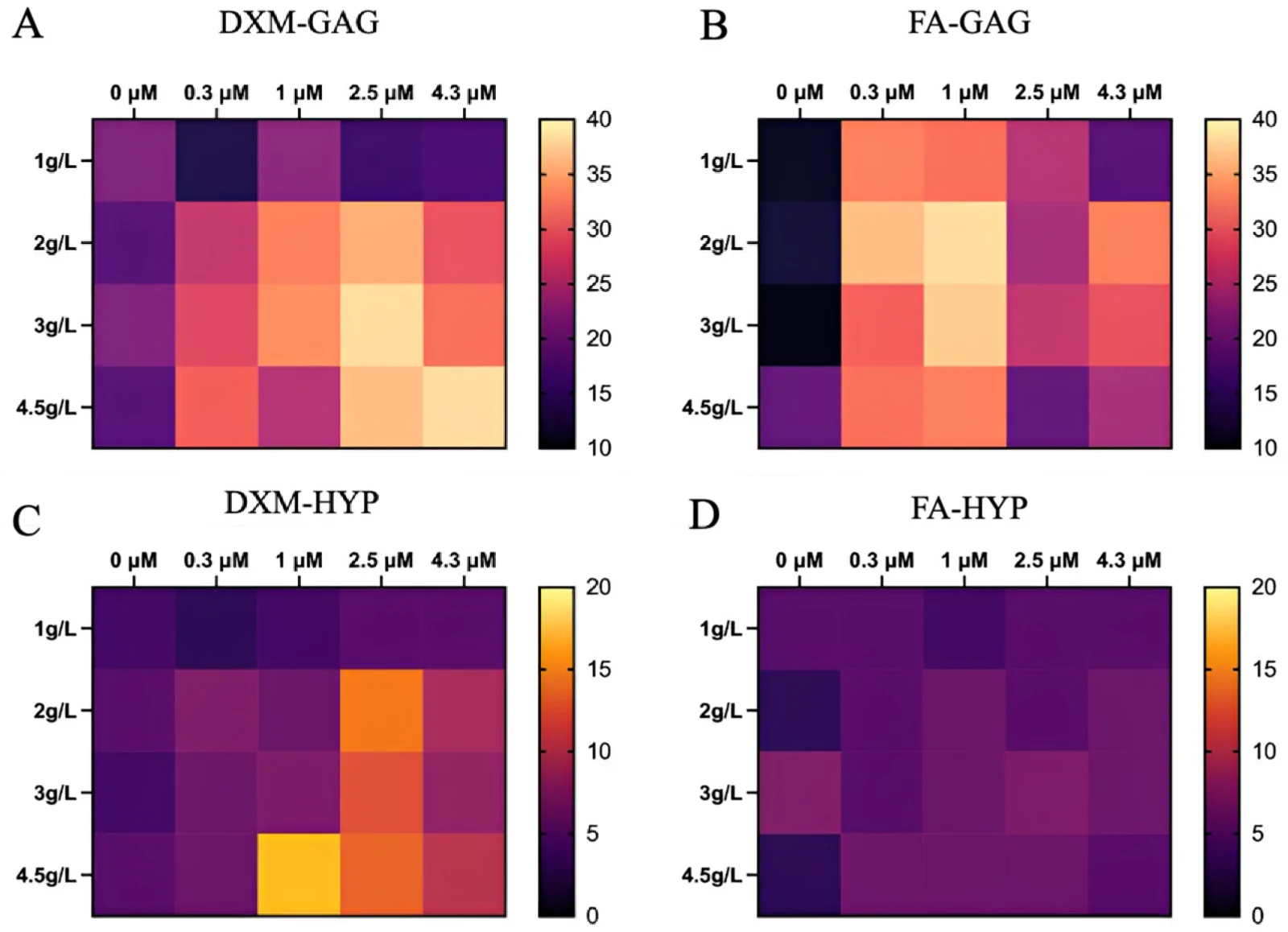
Heat map comparison of GAG and HYP content across experimental conditions (DXM and FA). Glucose concentration (g/L) and insulin level (µM) are represented on the y- and x-axes, respectively (n = 4).

Average levels of GAG and HYP contents for each treatment are presented in Figure 2. In DXM-treated aggregates, GAG was generally higher at higher glucose and insulin concentrations, while little GAG was detected at 1 g/L glucose without insulin. In FA-treated aggregates, higher GAG was observed primarily at 0.3 and 1 µM insulin, whereas little GAG was detected without insulin or at the highest insulin concentrations (Figure 2A,B). HYP in DXM-treated aggregates was generally higher at 3–4.5 g/L glucose, particularly at 1 and 2.5 µM insulin. In contrast, HYP remained low across most FA conditions (Figure 2C,D). For further insight into the separate DNA, GAG/DNA, and HYP/DNA values, see Supplementary Materials (Figures S7–S9).

### 3.3. Glucose Uptake

**Figure 3:**
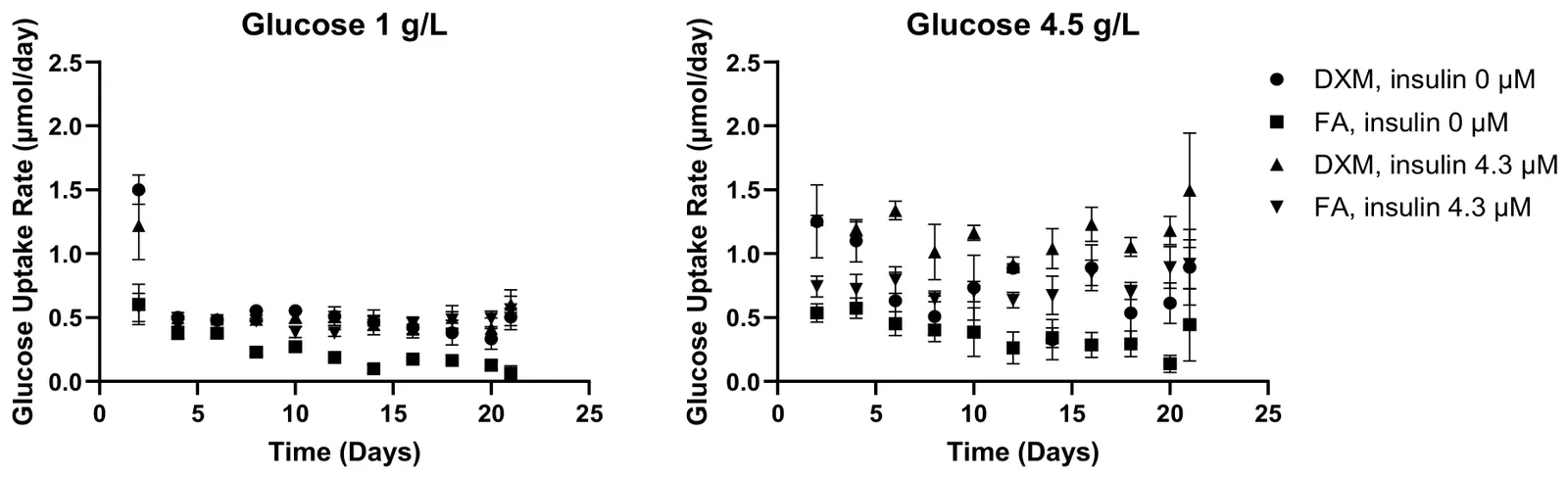
Temporal evolution of glucose uptake rate under DXM and FA as a function of insulin and glucose availability. Glucose uptake rates were determined throughout 21 days of differentiation under low (1 g/L; (**left**)) and high (4.5 g/L; (**right**)) initial glucose conditions (n = 3). Symbols denote corticosteroid and insulin conditions: DXM with 0 µM insulin (●), FA with 0 µM insulin (■), DXM with 4.3 µM insulin (▲), and FA with 4.3 µM insulin (▼). Glucose uptake exhibited significant time-dependent effects of corticosteroid, insulin, and glucose (time × corticosteroid, time × insulin, and time × glucose; all *p* < 0.001).

Cumulative glucose uptake increased throughout the 21-day culture period and differed between DXM- and FA-treated aggregates (see Supplementary Material, Figure S3). Subsequent analyses were based on interval-specific glucose uptake rates. Glucose uptake changed over time and differed between DXM and FA (Figure 3). DXM-treated aggregates generally had higher uptake rates early in culture. The effects of insulin and glucose became more apparent with time, with significant time × corticosteroid, time × insulin, and time × glucose interactions (all *p* < 0.001).

**Figure 4:**
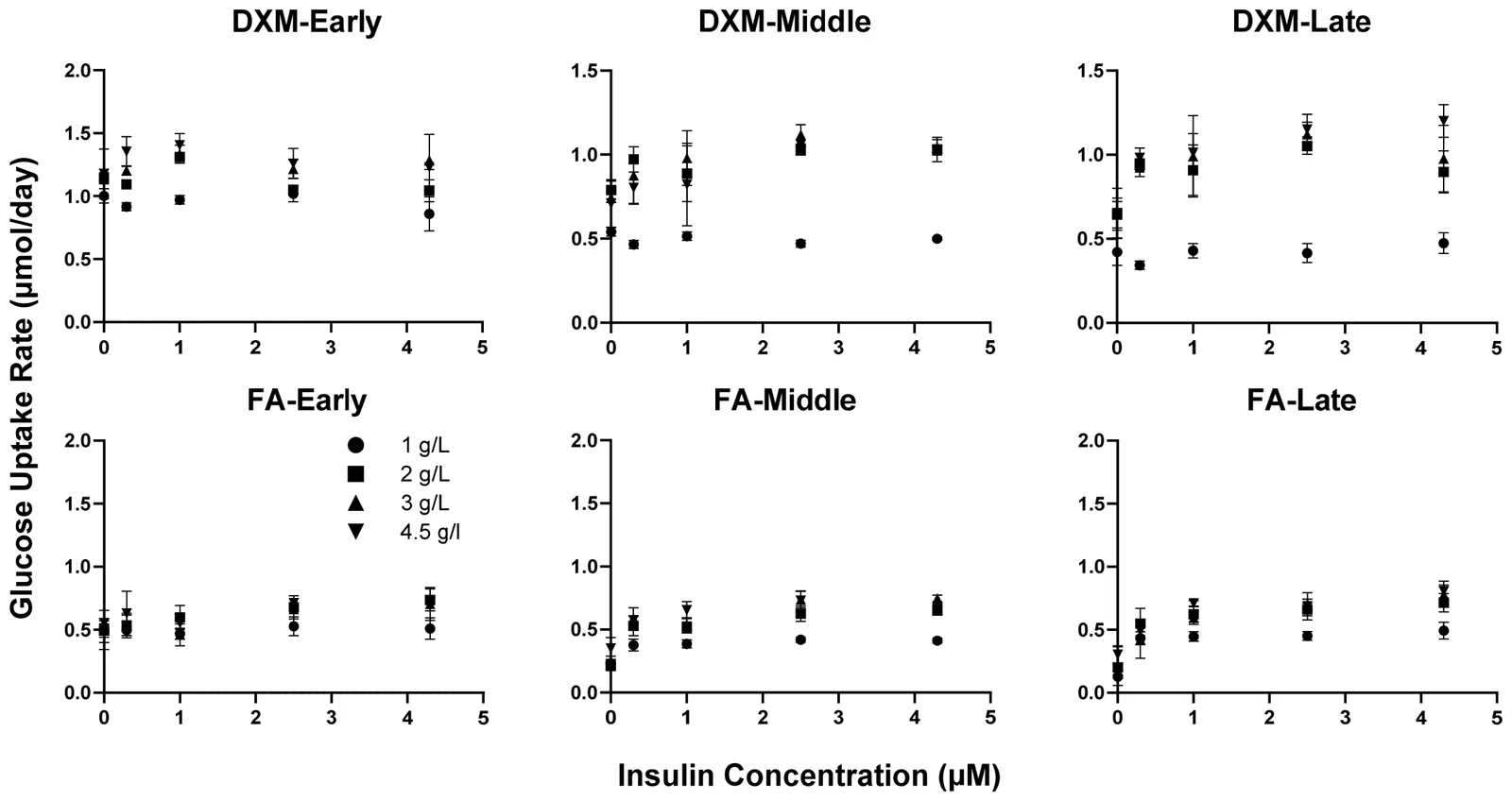
Insulin-dependent regulation of glucose uptake under DXM and FA during differentiation. Glucose uptake rates are shown as a function of insulin concentration during early (Days 0–4), mid (Days 6–12), and late (Days 12–21) culture for aggregates treated with DXM (**top row**) or FA (**bottom row**) (n = 3). Symbols denote initial glucose concentrations of 1 (●), 2 (■), 3 (▲), and 4.5 (▼) g/L. Longitudinal analysis demonstrated significant corticosteroid × insulin, insulin × glucose, and time × insulin interactions (all *p* < 0.001).

The effect of insulin also changed during differentiation (Figure 4). During early culture, glucose uptake varied relatively little with insulin concentration. During mid and late culture, uptake increased with insulin, particularly in FA-treated aggregates. By late culture, FA-treated aggregates without insulin had substantially lower glucose uptake than insulin-supplemented groups. Corticosteroid × insulin and insulin × glucose interactions were significant (both *p* < 0.001).

The effect of initial glucose concentration also varied with corticosteroid and insulin (see Supplementary Material, Figure S4). In DXM-treated aggregates, uptake generally increased between 1 and 2 g/L glucose, with smaller differences among the higher glucose concentrations. FA-treated aggregates showed a more gradual glucose response that varied with insulin concentration. Corticosteroid × glucose, insulin × glucose, and corticosteroid × insulin × glucose interactions were significant (all *p* < 0.001). Having established corticosteroid- and nutrient-dependent differences in glucose utilization, we next examined whether these metabolic differences were reflected in lactate secretion.

### 3.4. Lactate Secretion

**Figure 5:**
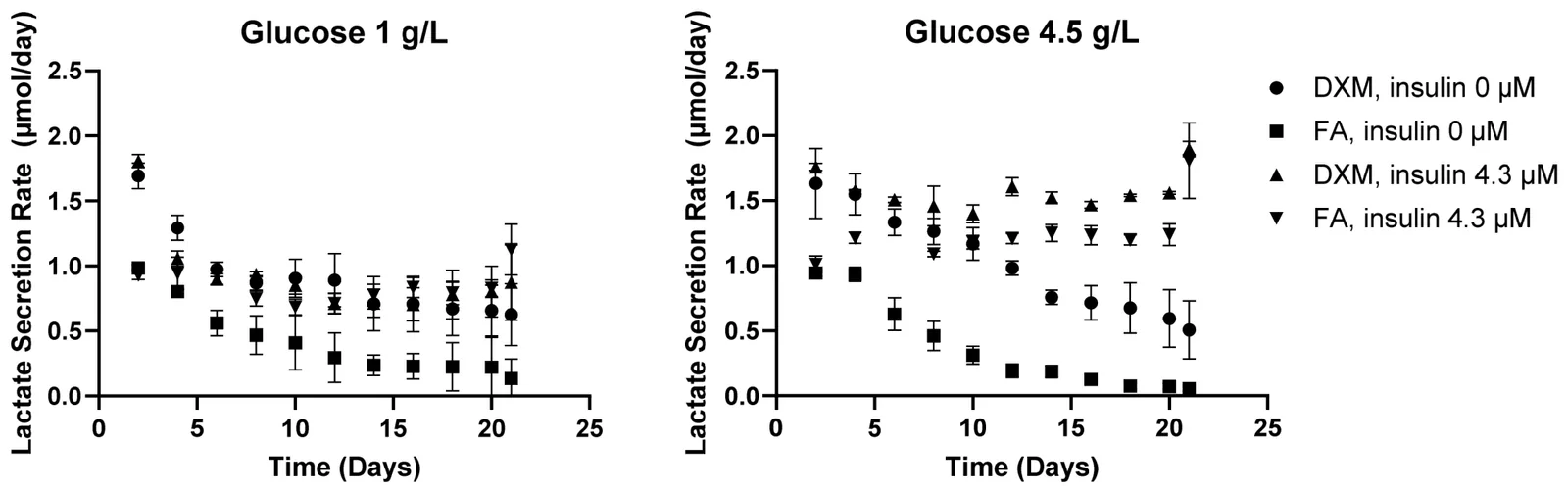
Temporal evolution of lactate secretion under DXM and FA as a function of insulin and glucose availability. Lactate secretion rates were measured throughout 21 days of differentiation under low (1 g/L; (**left**)) and high (4.5 g/L; (**right**)) initial glucose conditions (n = 3). Symbols denote corticosteroid and insulin conditions: DXM with 0 µM insulin (●), FA with 0 µM insulin (■), DXM with 4.3 µM insulin (▲), and FA with 4.3 µM insulin (▼). Lactate secretion exhibited significant time-dependent effects of corticosteroid, insulin, and glucose (time × corticosteroid, time × insulin, and time × glucose; all *p* < 0.001).

Cumulative lactate secretion increased throughout culture and was generally greater in DXM-treated than FA-treated aggregates (see Supplementary Material, Figure S5). Subsequent analyses were based on interval-specific lactate secretion rates. Lactate secretion changed over time and differed between DXM and FA (Figure 5). DXM-treated aggregates generally had higher secretion rates early in culture. The effects of insulin and glucose became more apparent with time, with significant time × corticosteroid, time × insulin, and time × glucose interactions (all *p* < 0.001).

**Figure 6:**
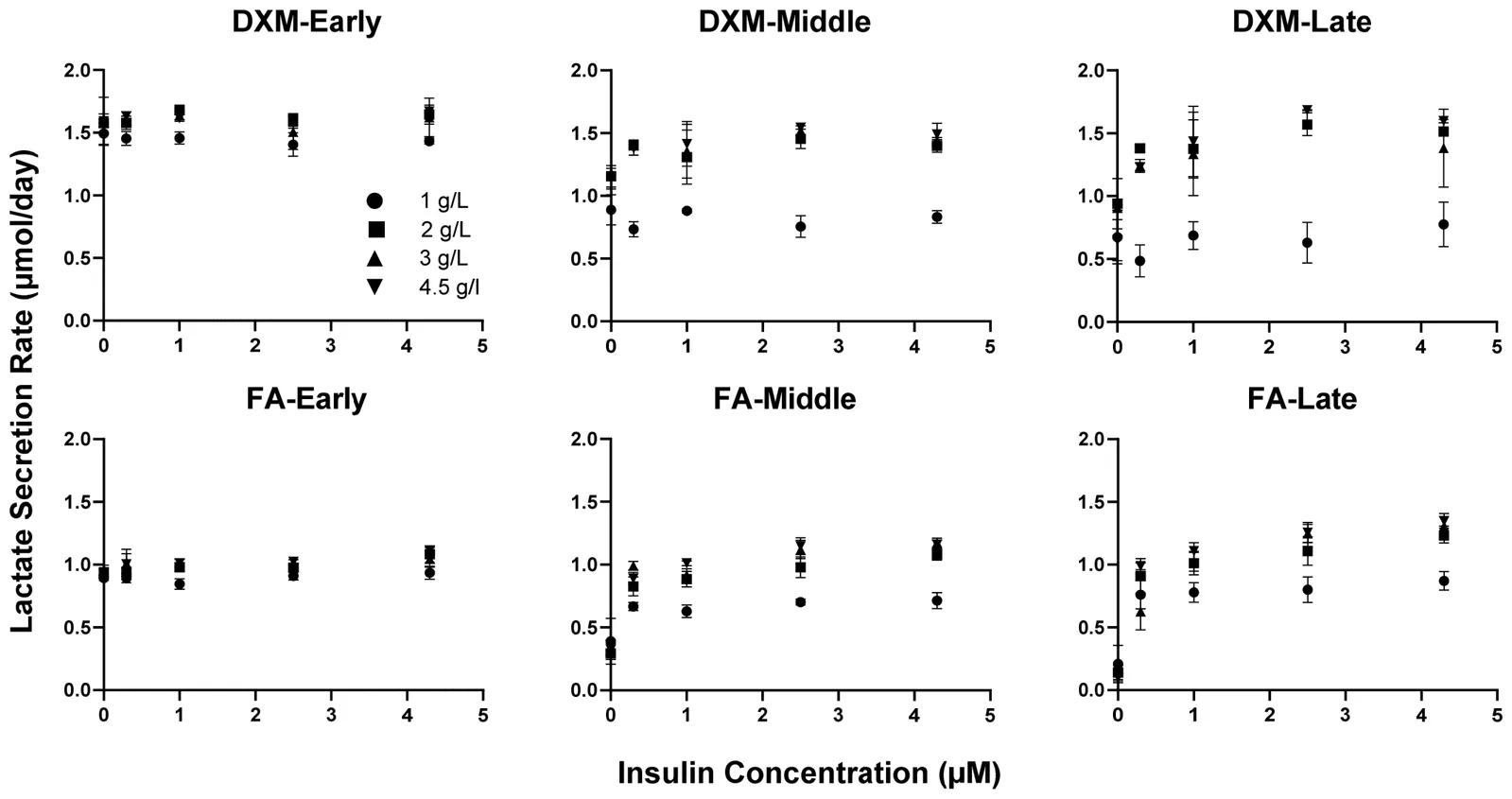
Insulin-dependent regulation of lactate secretion under DXM and FA during differentiation. Lactate secretion rates are shown as a function of insulin concentration during early (Days 0–4), mid (Days 6–12), and late (Days 12–21) culture for aggregates treated with DXM (**top row**) or FA (**bottom row**) (n = 3). Symbols denote initial glucose concentrations. Longitudinal statistical analysis demonstrated significant corticosteroid × insulin (*p* < 0.001), insulin × glucose (*p* < 0.001), and time × insulin (*p* < 0.001) interactions.

Insulin dependence increased during differentiation (Figure 6). During early culture, lactate secretion varied relatively little with insulin concentration. During mid and late culture, secretion increased with insulin, particularly in FA-treated aggregates. By late culture, FA-treated aggregates without insulin had substantially lower lactate secretion than insulin-supplemented groups. Corticosteroid × insulin and insulin × glucose interactions were significant (both *p* < 0.001).

The effect of glucose concentration also increased during differentiation (see Supplementary Material, Figure S6). DXM-treated aggregates showed a large increase in lactate secretion between 1 and 2 g/L glucose, with smaller differences at higher concentrations. FA-treated aggregates showed a more gradual response that varied with insulin concentration. Corticosteroid × glucose, insulin × glucose, and time × glucose interactions were significant (all *p* < 0.001).

### 3.5. Histology

**Figure 7:**
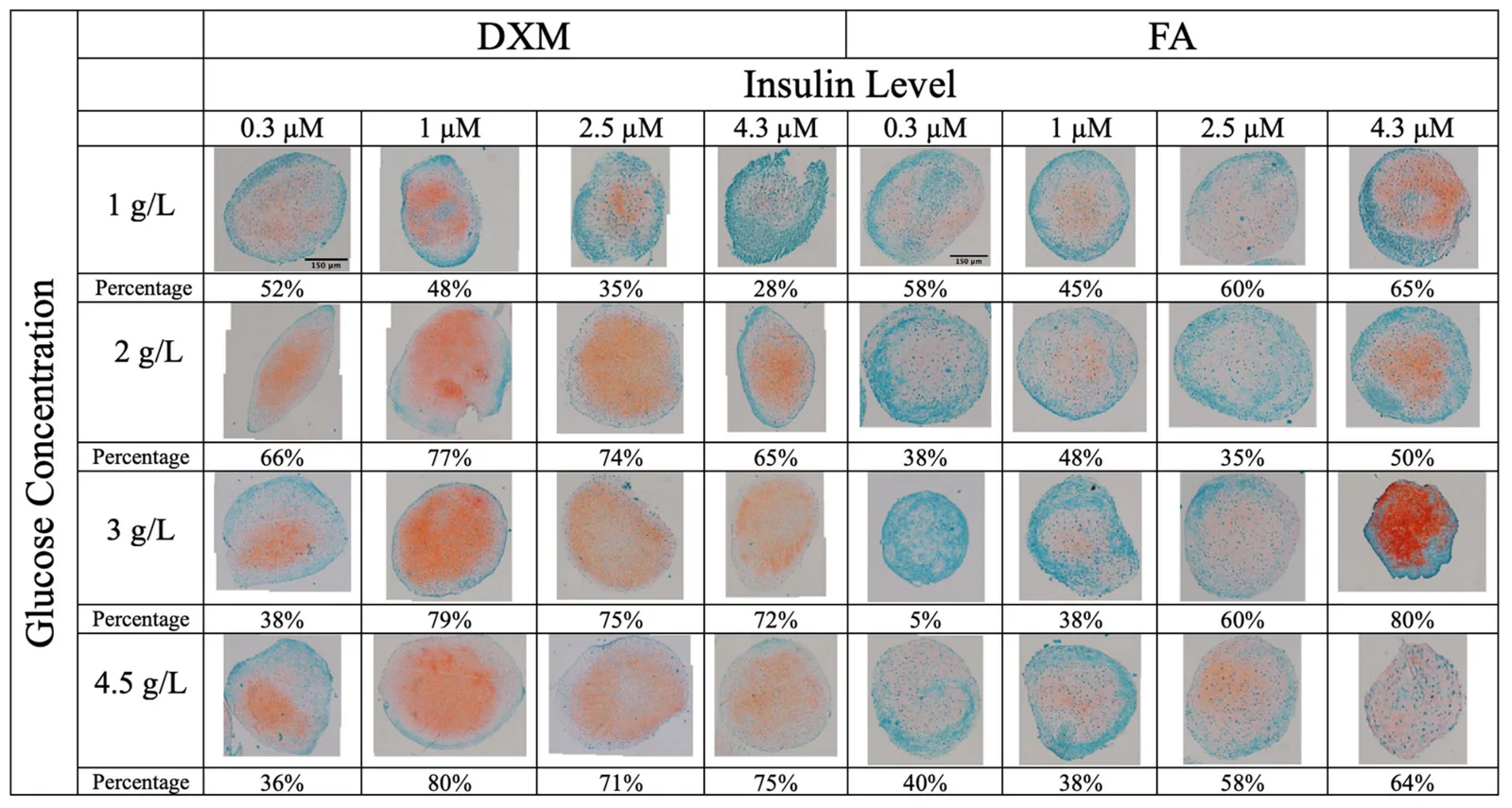
Histological assessment of aggregate cartilaginous matrix production via Safranin-O staining across varying corticosteroid type (DXM vs. FA), glucose concentration (1, 2, 3, and 4.5 g/L), and insulin level (0, 0.3, 1, 2.5, and 4.3 µM). Safranin-O staining appears orange-red, indicating the deposition of sulfated glycosaminoglycans (sGAGs) within the hMSC-derived chondrogenic aggregates, whereas green staining from the Fast Green counterstain highlights non-sGAG-rich tissue components, including collagenous extracellular matrix. Percentages beneath each image indicate the Safranin-O-positive area as a fraction of total aggregate area, determined by histomorphometry (n = 2–3). Scale bar = 150 μm.

Safranin O staining varied with corticosteroid, glucose, and insulin conditions. Quantitative analysis of the stained region (from light pink to dark orange) is also reported below each sample (Figure 7). In DXM-treated aggregates, stronger staining was generally observed at higher glucose concentrations, with the strongest staining at 1 µM insulin. Aggregates cultured at 1 g/L glucose showed relatively weak staining. In FA-treated aggregates, stronger Safranin O staining was observed primarily at higher insulin concentrations, particularly 4.3 µM. FA-43 (4.3 µM insulin and 3 g/L glucose) showed especially strong staining. Additional FA replicates at 2.5 and 4.3 µM insulin are shown in Supplementary Material, Figure S2. To allow comparison of samples from both groups, additional replicates of the DXM-treated samples at insulin levels of 2.5 µM and 4.3 µM are also provided in Figure S2.

### 3.6. Immunohistochemistry

**Figure 8:**
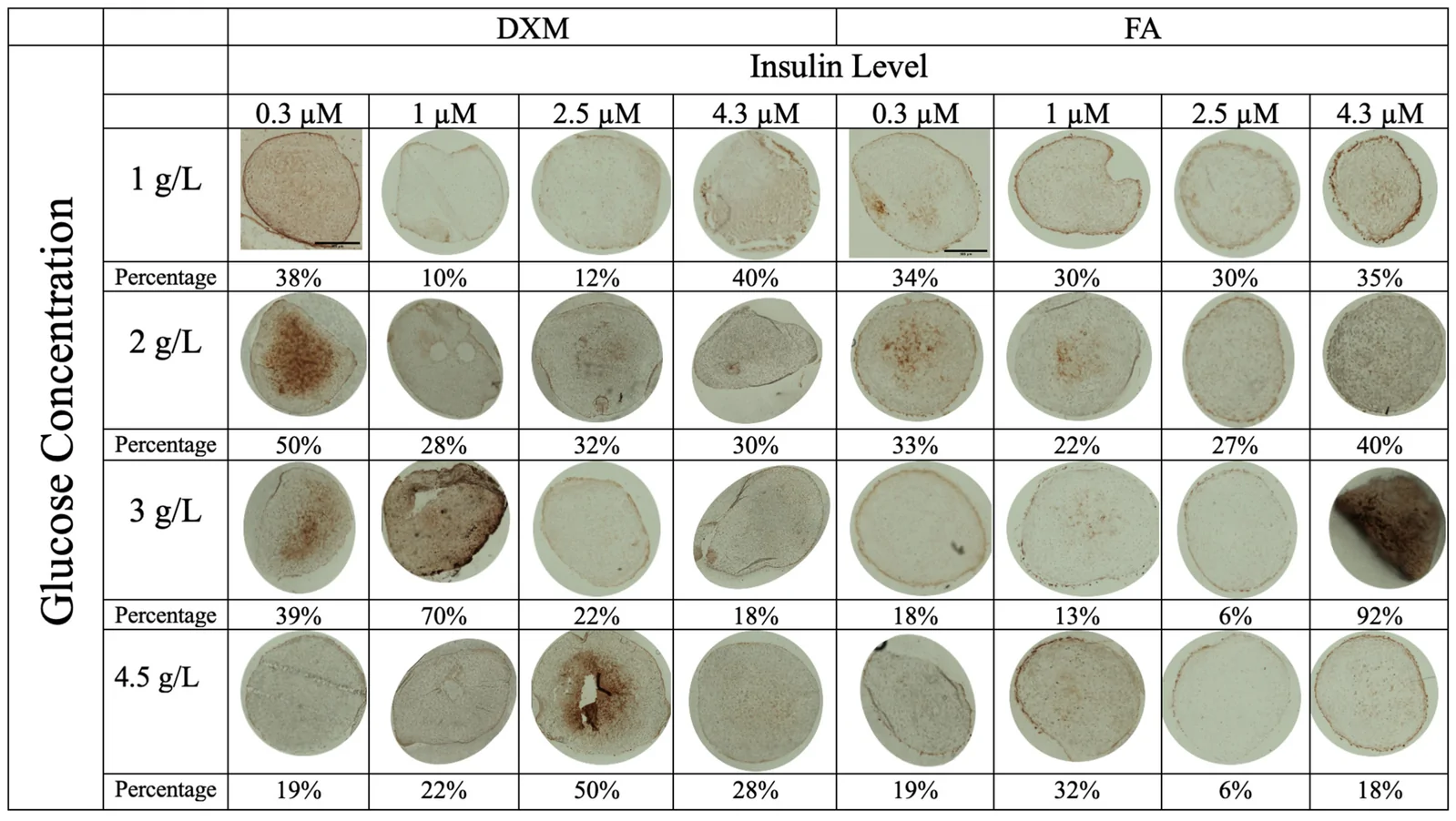
Immunohistochemical assessment of collagen type II staining in chondrogenic aggregates across varying corticosteroid type (DXM vs. FA), glucose concentration (1, 2, 3, and 4.5 g/L), and insulin level (0, 0.3, 1, 2.5, and 4.3 µM). Brown to reddish-brown staining indicates positive immunoreactivity for collagen II, reflecting the presence and spatial distribution of the respective collagen proteins within the hMSC-derived chondrogenic aggregates. Percentages beneath each image indicate the collagen II-positive area as a fraction of total aggregate area, determined by histomorphometry (n = 2–3). Scale bar = 300 μm.

Collagen II staining was detected in all treatment groups (n = 2–3), although its intensity and distribution varied among conditions (Figure 8). In DXM-treated aggregates, the strongest staining was observed at 1 µM insulin and 3 g/L glucose. In FA-treated aggregates, staining was primarily peripheral, with the strongest staining observed in FA-43 (4.3 µM insulin and 3 g/L glucose).

**Figure 9:**
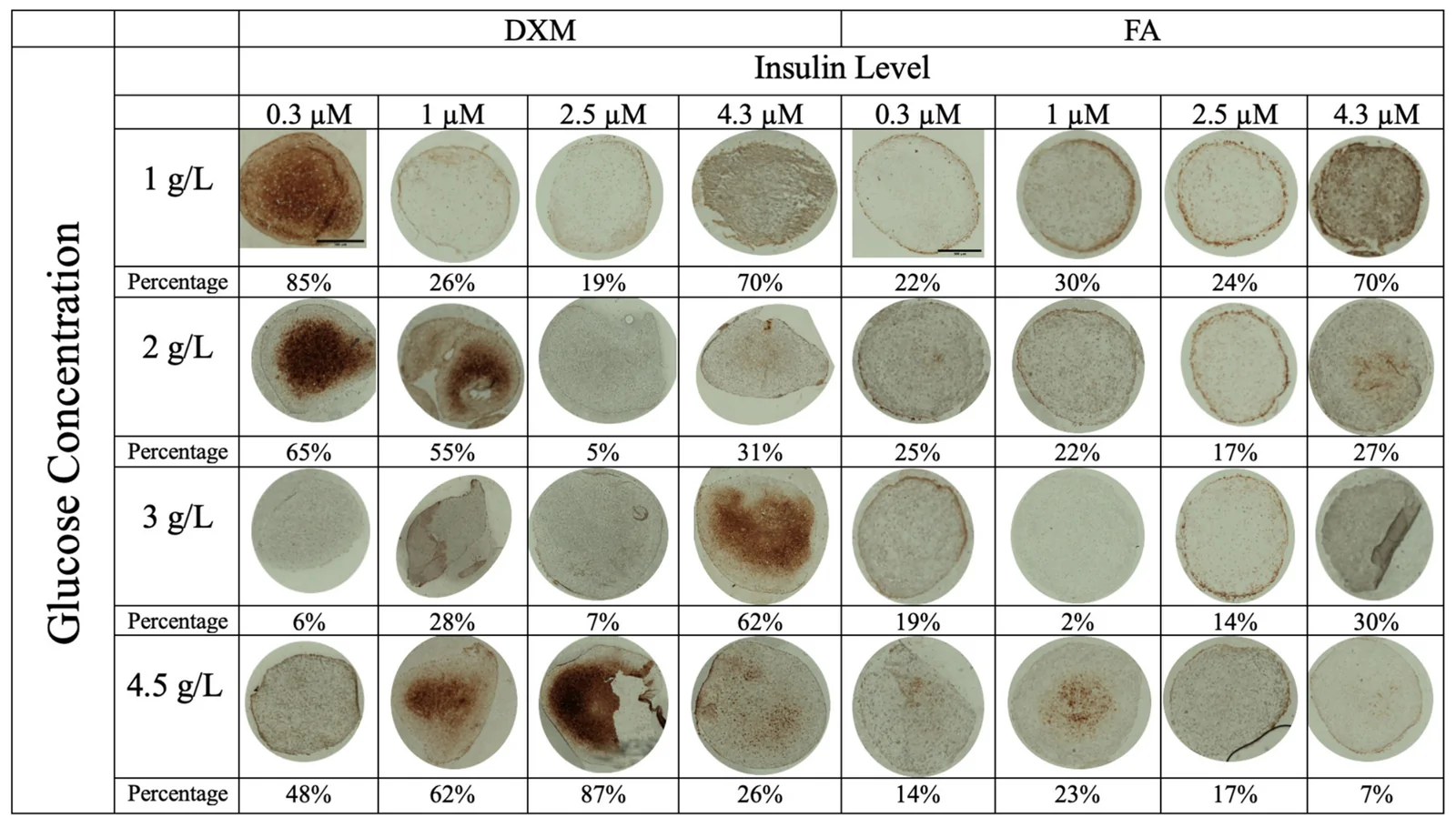
Immunohistochemical assessment of collagen type X staining in chondrogenic aggregates across varying corticosteroid type (DXM vs. FA), glucose concentration (1, 2, 3, and 4.5 g/L), and insulin level (0, 0.3, 1, 2.5, and 4.3 µM). Brown to reddish-brown staining indicates positive immunoreactivity for collagen X, reflecting the presence and spatial distribution of the respective collagen proteins within the hMSC-derived chondrogenic aggregates. Percentages beneath each image indicate the collagen X-positive area as a fraction of total aggregate area, determined by histomorphometry (n = 2–3). Scale bar = 300 μm.

Collagen X staining differed substantially between DXM- and FA-treated aggregates (Figure 9). Positive staining was observed in approximately half of the DXM conditions and was generally localized to the aggregate interior. In contrast, most FA-treated aggregates showed little or no collagen X staining, with detectable staining limited primarily to selected conditions at 4.3 µM insulin.

## 4. Discussion

This study demonstrates that corticosteroid identity, insulin concentration, and glucose availability interact to regulate hMSC chondrogenesis in TGF-β1-containing medium. DXM and FA produced distinct phenotypes. FA-treated aggregates were generally smaller and more compact and, under selected high-insulin conditions, exhibited strong Safranin O and collagen II staining with little detectable collagen X. In contrast, DXM-treated aggregates were larger and more irregular and showed collagen X staining under several conditions. These findings support further investigation of FA as an alternative to DXM for promoting a cartilage-like phenotype while limiting hypertrophic differentiation. These corticosteroid-dependent differences are consistent with previous studies showing that glucocorticoid effects on MSC chondrogenesis depend strongly on the growth factor and culture microenvironment [4,28].

DXM and FA also produced distinct metabolic responses. Glucose uptake and lactate secretion were generally higher under DXM, particularly during early culture, whereas insulin and glucose dependence became more apparent as differentiation progressed. This was especially evident under FA: during later stages, glucose uptake and lactate secretion increased with insulin supplementation and were substantially lower without insulin. Insulin is important for MSC chondrogenesis, and its effects are concentration dependent, with marker expression increasing up to approximately 10 µg/mL and plateauing at 50 µg/mL [13]. Our highest concentration (4.3 µM, approximately 25 µg/mL) exceeds typical ITS levels but was chosen deliberately as a high-insulin condition and lies within this studied range. However, our results further suggest that the effect of insulin depends on corticosteroid identity and changes during differentiation.

Glucose concentration also affected DXM- and FA-treated aggregates differently. Under DXM, glucose uptake and lactate secretion increased substantially between 1 and 2 g/L glucose, with smaller differences at higher concentrations. FA-treated aggregates showed a more gradual glucose response that depended strongly on insulin concentration. Previous studies from our group similarly showed that glucose availability alters glucose uptake, lactate secretion, and matrix synthesis during hMSC chondrogenesis [14,24]. The present findings extend these observations by showing that the response to glucose also depends on corticosteroid identity and its interaction with insulin.

Interestingly, the metabolic differences did not directly correspond to the observed cartilage-like phenotype. DXM-treated aggregates generally consumed more glucose and produced more lactate, whereas FA-treated aggregates maintained lower metabolic rates despite their compact morphology and, under selected conditions, strong cartilage-matrix staining. Chondrogenic differentiation is associated with substantial changes in glucose metabolism and a shift toward a predominantly glycolytic phenotype [24,29], and changes in glycolysis have also been associated with chondrogenic maturation and hypertrophy [30]. Thus, greater glucose uptake and lactate production were not necessarily associated with a more favorable cartilage-like phenotype.

Histologic and biochemical measurements were not fully concordant. Safranin O staining was particularly strong in selected high-insulin FA conditions, whereas biochemical GAG measurements did not show a corresponding increase. This may reflect aggregate size, spatial variation in matrix deposition, section location, or normalization. Histology reflects local matrix distribution and density, whereas biochemical assays measure total recovered GAG and HYP. Thus, FA may affect the distribution or local density of deposited matrix without proportionally increasing total GAG content.

A potential mechanism for the FA-associated phenotype involves interactions among glucocorticoid, TGF-β, and insulin signaling. Hara et al. reported that FA enhanced TGF-β3-associated Smad2/3 and AKT/mTORC1 signaling in hMSCs [10]. Our findings in a TGF-β1-based system suggest that the effects of FA may not be limited to TGF-β3. Because insulin signaling also involves AKT, interactions among these pathways could contribute to the increasing insulin dependence observed under FA. Direct measurements of these signaling pathways will be needed to test this mechanism.

The limited collagen X staining in FA-treated aggregates is particularly relevant because hypertrophic differentiation remains a major challenge in hMSC-based cartilage tissue engineering [6,20]. FA-treated aggregates showed collagen II and proteoglycan staining under selected conditions while exhibiting substantially less collagen X staining than DXM-treated aggregates. Because collagen X staining was assessed qualitatively, additional hypertrophic markers, including *COL10A1*, *RUNX2*, alkaline phosphatase, and matrix mineralization, will be needed to determine whether FA consistently reduces hypertrophic differentiation.

This study has limitations. All experiments used cells from a single donor, so the reported effects reflect this cell source, and donor-to-donor variability in differentiation capacity may influence their absolute magnitude. In addition, the histologic and immunohistochemical assessments were primarily qualitative. Future studies should include additional donors, quantitative matrix analysis, and mechanical characterization, and more focused evaluation of insulin and glucose concentrations around the most favorable FA conditions.

Overall, FA produced a distinct cartilage-like and metabolic phenotype compared with DXM. FA-treated aggregates were more compact, showed favorable matrix staining under selected insulin conditions, and exhibited limited collagen X staining. Their metabolic activity also became increasingly insulin-dependent during differentiation. Together, these findings show that corticosteroid selection interacts with the metabolic composition of the differentiation medium and support further investigation of FA as an alternative corticosteroid for cartilage tissue engineering.

## 5. Conclusions

This study showed that FA and DXM produced distinct chondrogenic responses in hMSC aggregates under varying glucose and insulin conditions. FA-treated aggregates generally exhibited a smaller, more compact morphology, with pronounced Safranin O and collagen II staining and limited collagen X staining under selected high-insulin conditions. DXM-treated aggregates were generally larger and more irregular, with collagen X staining observed under some low- and high-glucose conditions. These findings suggest that insulin may enhance FA-mediated cartilage matrix formation, whereas glucose availability may influence the response to DXM and FA differently. Overall, the results support further investigation of FA as an alternative corticosteroid and highlight the importance of optimizing insulin and glucose concentrations in defined chondrogenic media.

## Figures and Tables

**Table 1:** Experimental groups for hMSC chondrogenic differentiation treated with DXM across varying glucose and insulin concentrations.

	Insulin Concentration	0 µM	0.3 µM	1 µM	2.5 µM	4.3 µM
Initial Glucose Level						
1 g/L		DXM 01	DXM 11	DXM 21	DXM 31	DXM 41
2 g/L		DXM 02	DXM 12	DXM 22	DXM 32	DXM 42
3 g/L		DXM 03	DXM 13	DXM 23	DXM 33	DXM 43
4.5 g/L		DXM 04	DXM 14	DXM 24	DXM 34	DXM 44

**Table 2:** Experimental groups for hMSC chondrogenic differentiation treated with FA across varying glucose and insulin concentrations.

	Insulin Concentration	0 µM	0.3 µM	1 µM	2.5 µM	4.3 µM
Initial Glucose Level						
1 g/L		FA 01	FA 11	FA 21	FA 31	FA 41
2 g/L		FA 02	FA 12	FA 22	FA 32	FA 42
3 g/L		FA 03	FA 13	FA 23	FA 33	FA 43
4.5 g/L		FA 04	FA 14	FA 24	FA 34	FA 44

## Data Availability

The original contributions presented in this study are included in the article/Supplementary Material. Further inquiries can be directed to the corresponding author.

## Supplementary Materials

The following supporting information can be downloaded at: https://www.mdpi.com/article/10.3390/cells15212034/s1. Table S1. Aggregate diameter (mm) presented as mean ± standard deviation (SD) (N = 8), Table S2. Circularity of aggregates shown as mean ± standard deviation (SD) (N = 8), Figure S1. Gross morphology of chondrogenic aggregates at Day 21 under stereomicroscopy across varying corticosteroid type (DXM vs. FA), glucose concentration (1, 2, 3, and 4.5 g/L), and insulin level (0 µM). Scale bar = 0.5 mm, Figure S2. Safranin O staining of additional DXM and FA-treated aggregates cultured at 2.5 and 4.3 µM insulin across the four initial glucose concentrations. Scale bar = 50 µm, Figure S3. Cumulative glucose uptake during differentiation under DXM and FA. Cumulative glucose uptake was measured throughout 21 days of differentiation under varying initial glucose and insulin concentrations in aggregates treated with DXM or FA, Figure S4. Glucose availability-dependent regulation of glucose uptake under DXM and FA during differentiation. Glucose uptake rates are shown as a function of initial glucose concentration during early (Days 0–4), mid (Days 6–12), and late (Days 12–21) culture for aggregates treated with DXM (top row) or FA (bottom row). Symbols denote insulin concentrations of 0 (●), 0.3 (■), 1 (▲), 2.5 (▼), and 4.3 (◆) µM. Longitudinal analysis demonstrated significant corticosteroid × glucose, insulin × glucose, and time × glucose interactions (all *p* < 0.001), Figure S5. Temporal profiles of cumulative lactate secretion across corticosteroid type (DXM vs. FA) and varying glucose and insulin conditions over a 21-day culture period. Panel A and Panel B represent DXM- and FA-treated aggregates, respectively. Glucose concentration was tested at 1, 2, 3, and 4.5 g/L, and insulin level at 0, 0.3, 1, 2.5, and 4.3 µM, Figure S6. Glucose-dependent regulation of lactate secretion under DXM and FA during differentiation. Lactate secretion rates are shown as a function of initial glucose concentration during early (Days 0–4), mid (Days 6–12), and late (Days 12–21) culture for aggregates treated with DXM (top row) or FA (bottom row). Symbols denote insulin concentrations of 0 (●), 0.3 (■), 1 (▲), 2.5 (▼), and 4.3 (◆) µM. Longitudinal statistical analysis demonstrated significant corticosteroid × glucose, insulin × glucose, and time × glucose interactions (all *p* < 0.001), Figure S7. DNA content (ng) of DXM-treated aggregates at day 21. Data are presented as mean ± SD (N = 4), Figure S8. DNA content (ng) of FA-treated aggregates at day 21. Data are presented as mean ± SD (N = 4), Figure S9. Heat map comparison of normalized GAG and HYP content across experimental conditions. (A,B) DNA-normalized GAG content in DXM- and FA-treated aggregates, respectively. (C,D) DNA-normalized HYP content normalized in DXM- and FA-treated aggregates, respectively. Glucose concentration (g/L) is shown on the y-axis and insulin concentration (µM) on the y-axis. Color indicates the mean normalized value, with the scale shown in each panel.

## Abbreviations

DXM	Dexamethasone
FA	Fluocinolone Acetonide
hMSCs	human Mesenchymal Stem Cells
ITS	Insulin–Transferrin–Selenium
GAGs	Glycosaminoglycans
HYP	Hydroxyproline
ECM	Extracellular Matrix
IHC	Immunohistochemistry
GEE	Generalized Estimating Equations
