# Supplementary material for: Comparative Chondrogenic Efficacy of Dexamethasone Versus Fluocinolone Acetonide Under Varying Insulin and Glucose Concentrations in Defined Media
Source: Cells. 2026 Nov 7;15(21):2034. doi: 10.3390/cells15212034 (PMC13761659; doi:10.3390/cells15212034)
Supplement: Supplementary file 1 [file cells-15-02034-s001.zip › cells-4574731-supplementary.pdf]

Supplementary Materials

Comparative Chondrogenic Efficacy of Dexamethasone Versus Fluocinolone Acetonide Under Varying Insulin and Glucose Concentrations in Defined Media

Bo-An Chen, Nima Firouzi, Rodrigo A. Somoza and Harihara Baskaran

Table S1. Aggregate diameter (mm) presented as mean ± standard deviation (SD) (N=8)

		DXM					FA				
		Insulin Level									
		0 μM	0.3 μM	1 μM	2.5 μM	4.3 μM	0 μM	0.3 μM	1 μM	2.5 μM	4.3 μM
Glucose Concentration	1g/L	1.08 ± 0.26	0.97 ± 0.06	1.06 ± 0.06	1 ± 0.03	1.14 ± 0.10	0.58 ± 0.05	1.08 ± 0.04	1.08 ± 0.04	1.1 ± 0.03	1.14 ± 0.05
	2g/L	1.98 ± 0.23	1.9 ± 0.15	2.18 ± 0.24	2.72 ± 0.14	2.62 ± 0.13	0.5 ± 0.03	1.08 ± 0.06	1.18 ± 0.02	1.18 ± 0.07	1.22 ± 0.07
	3g/L	1.74 ± 0.19	1.82 ± 0.22	1.96 ± 0.23	2.48 ± 0.32	2.36 ± 0.29	0.48 ± 0.02	0.86 ± 0.02	1.12 ± 0.3	1.3 ± 0.02	1.3 ± 0.02
	4.5 g/L	1.3 ± 0.19	1.68 ± 0.11	2.4 ± 0.33	2.86 ± 0.19	2.32 ± 0.18	0.46 ± 0.01	1.04 ± 0.36	1.08 ± 0.02	1.26 ± 0.06	1.24 ± 0.07

Table S2. Circularity of aggregates shown as mean ± standard deviation (SD) (N=8)

		DXM					FA				
		Insulin Level									
		0 μM	0.3 μM	1 μM	2.5 μM	4.3 μM	0 μM	0.3 μM	1 μM	2.5 μM	4.3 μM
Glucose Concentration	1g/L	0.62 ± 0.25	0.91 ± 0.00	0.92 ± 0.02	0.91 ± 0.03	0.91 ± 0.02	0.93 ± 0.01	0.93 ± 0.01	0.94 ± 0.00	0.95 ± 0.01	0.95 ± 0.00
	2g/L	0.69 ± 0.19	0.84 ± 0.07	0.91 ± 0.03	0.93 ± 0.01	0.89 ± 0.05	0.91 ± 0.01	0.94 ± 0.00	0.95 ± 0.01	0.94 ± 0.02	0.96 ± 0.00
	3g/L	0.57 ± 0.20	0.87 ± 0.06	0.91 ± 0.04	0.89 ± 0.04	0.86 ± 0.09	0.77 ± 0.34	0.95 ± 0.00	0.95 ± 0.00	0.95 ± 0.00	0.96 ± 0.00
	4.5 g/L	0.68 ± 0.18	0.90 ± 0.03	0.88 ± 0.04	0.91 ± 0.04	0.93 ± 0.03	0.92 ± 0.01	0.94 ± 0.01	0.95 ± 0.00	0.96 ± 0.00	0.95 ± 0.01

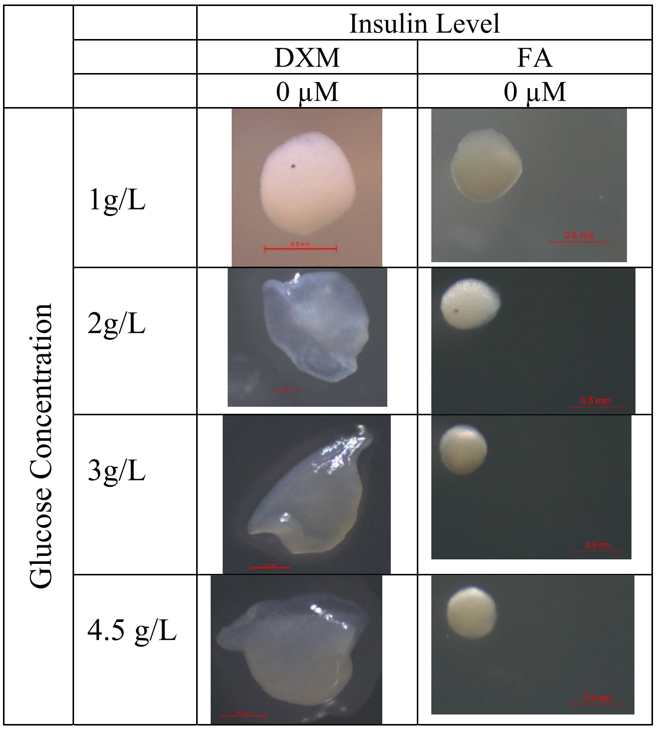

Fig S1. Gross morphology of chondrogenic aggregates at Day 21 under stereomicroscopy across varying corticosteroid type (DXM vs. FA), glucose concentration (1, 2, 3, and 4.5 g/L), and insulin level (0 μM). Scale bar = 0.5 mm.

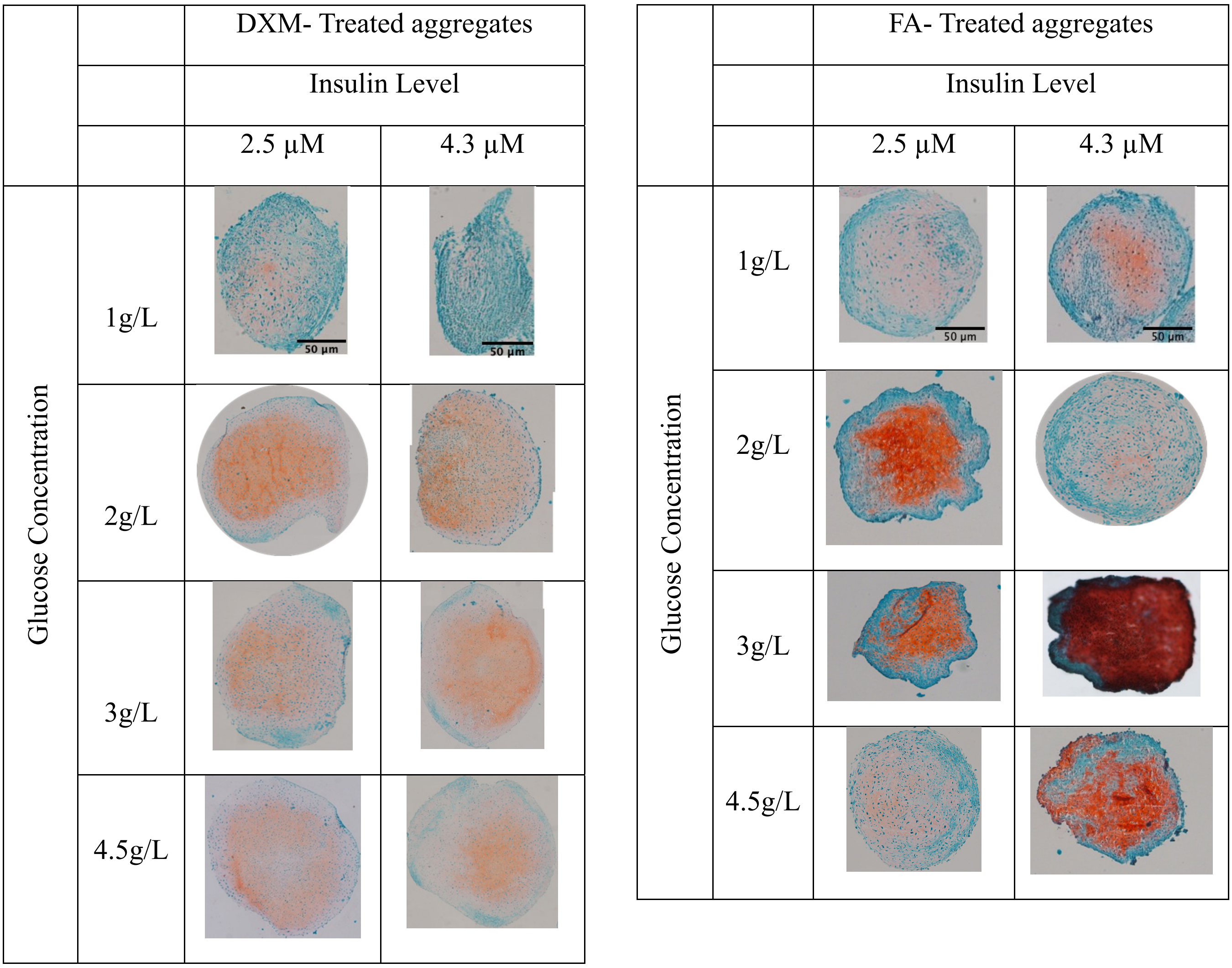

Fig. S2. Safranin O staining of additional DXM and FA-treated aggregates cultured at 2.5 and 4.3 μM insulin across the four initial glucose concentrations. Scale bar = 50 μm.

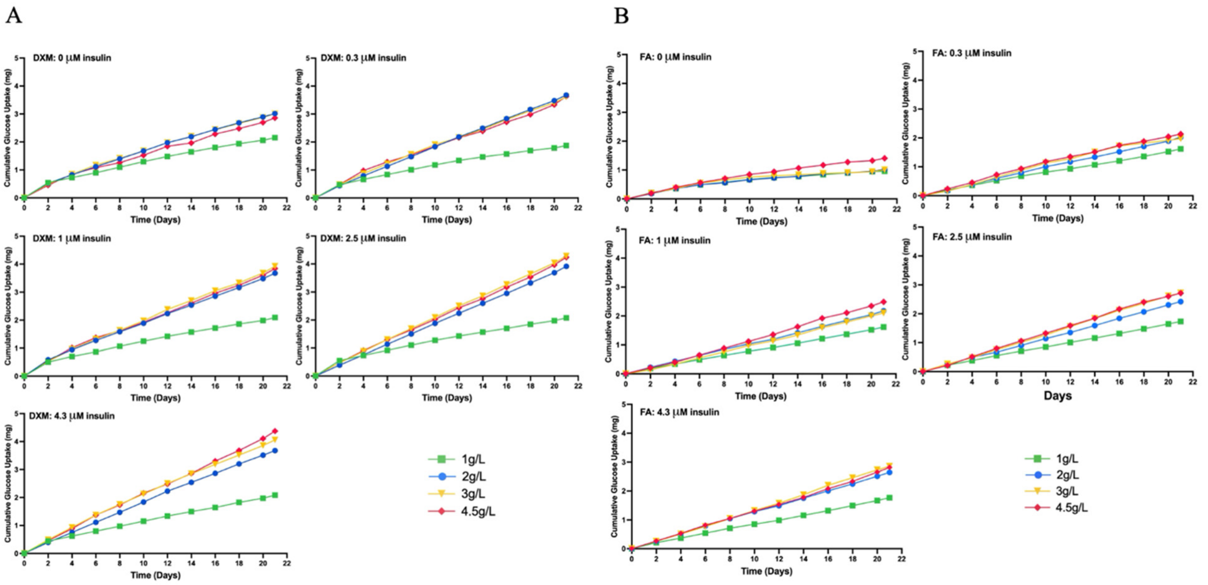

Fig. S3. Cumulative glucose uptake during differentiation under DXM and FA. Cumulative glucose uptake was measured throughout 21 days of differentiation under varying initial glucose and insulin concentrations in aggregates treated with DXM or FA.

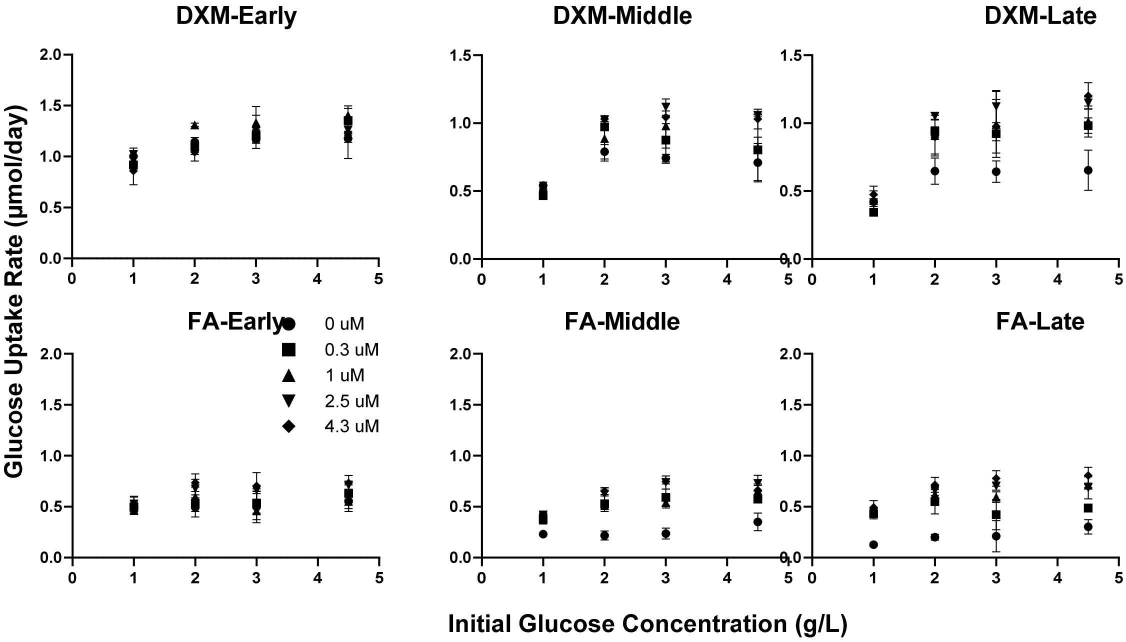

Fig. S4. Glucose availability-dependent regulation of glucose uptake under DXM and FA during differentiation. Glucose uptake rates are shown as a function of initial glucose concentration during early (Days 0–4), mid (Days 6–12), and late (Days 12–21) culture for aggregates treated with DXM (top row) or FA (bottom row). Symbols denote insulin concentrations of 0 (●), 0.3 (■), 1 (▲), 2.5 (▼), and 4.3 (◆) μM. Longitudinal analysis demonstrated significant corticosteroid × glucose, insulin × glucose, and time × glucose interactions (all $p < 0.001$).

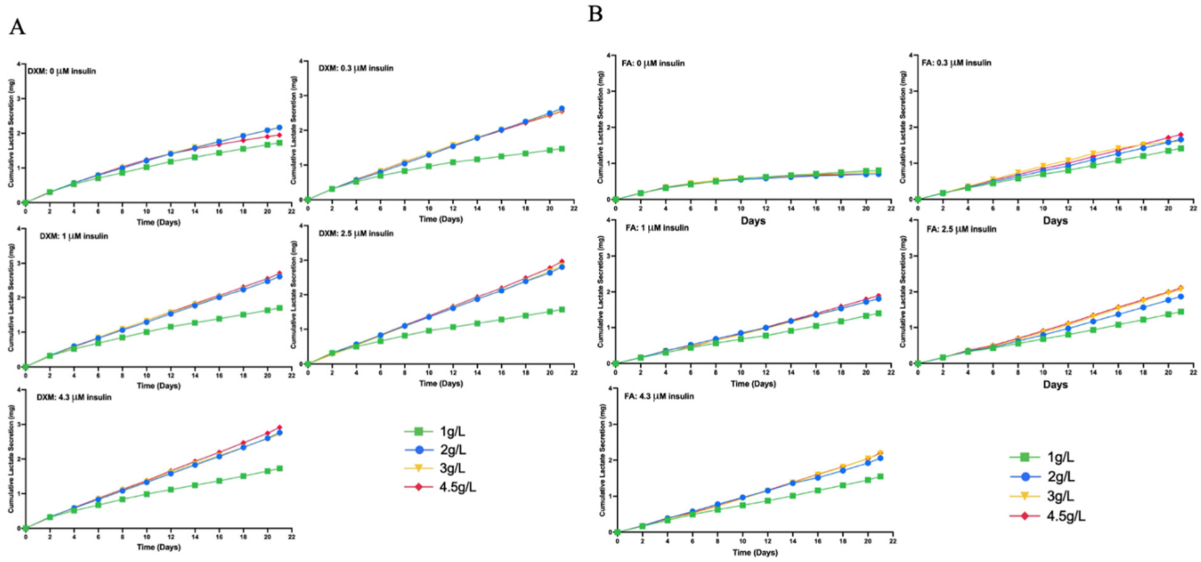

Fig S5. Temporal profiles of cumulative lactate secretion across corticosteroid type (DXM vs. FA) and varying glucose and insulin conditions over a 21-day culture period. Panel A and Panel B represent DXM- and FA-treated aggregates, respectively. Glucose concentration was tested at 1, 2, 3, and 4.5 g/L, and insulin level at 0, 0.3, 1, 2.5, and 4.3 μM.

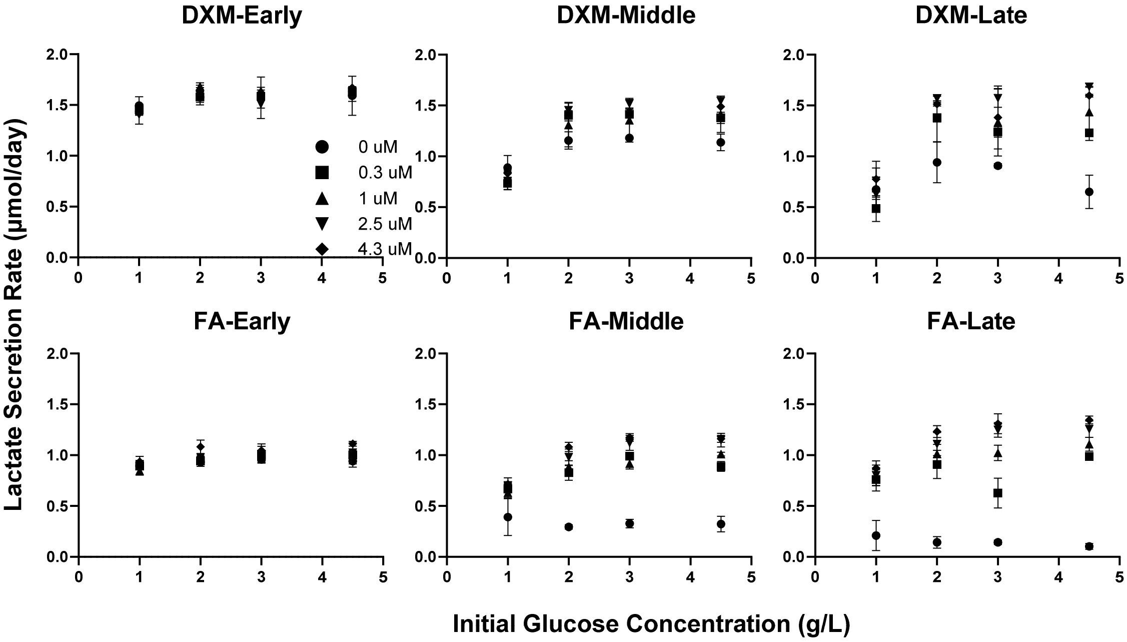

Fig. S6. Glucose-dependent regulation of lactate secretion under DXM and FA during differentiation. Lactate secretion rates are shown as a function of initial glucose concentration during early (Days 0–4), mid (Days 6–12), and late (Days 12–21) culture for aggregates treated with DXM (top row) or FA (bottom row). Symbols denote insulin concentrations of 0 (●), 0.3 (■), 1 (▲), 2.5 (▼), and 4.3 (◆) µM. Longitudinal statistical analysis demonstrated significant corticosteroid × glucose, insulin × glucose, and time × glucose interactions (all $p < 0.001$).

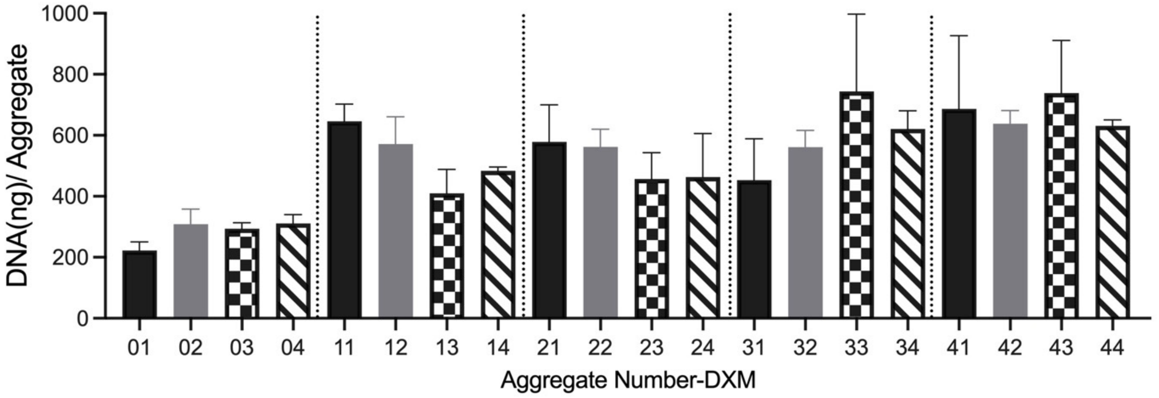

Fig. S7. DNA content (ng) of DXM-treated aggregates at day 21. Data are presented as mean ± SD (N = 4).

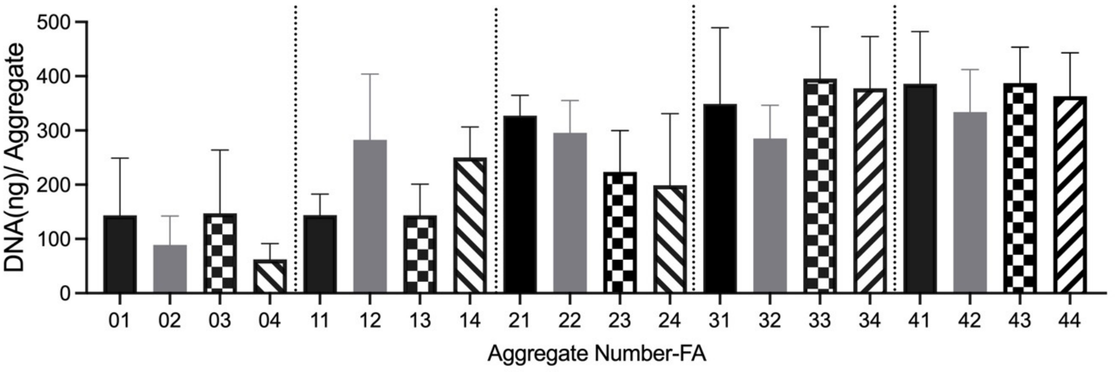

Fig. S8. DNA content (ng) of FA-treated aggregates at day 21. Data are presented as mean ± SD (N = 4).

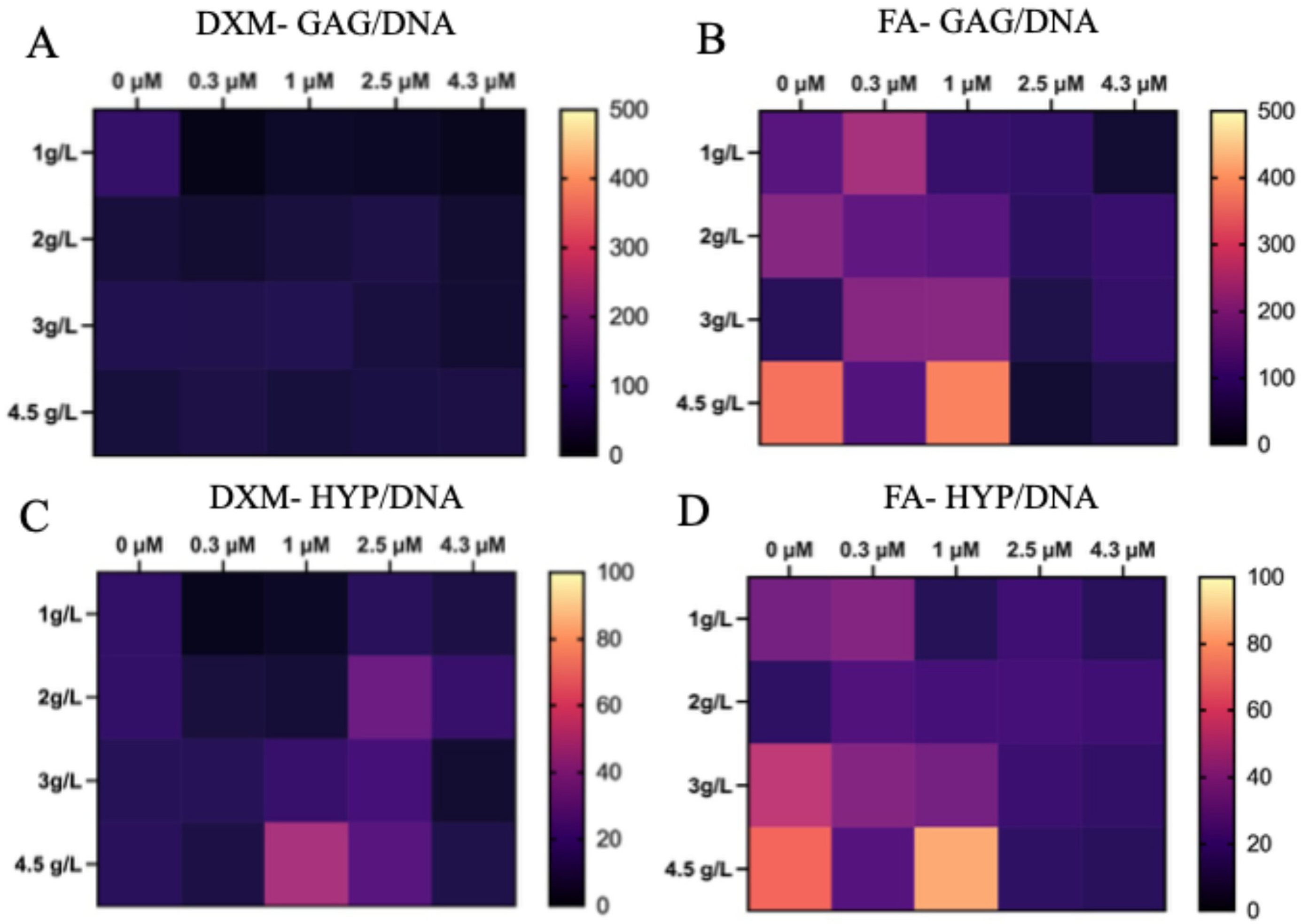

Fig S9. Heat map comparison of normalized GAG and HYP content across experimental conditions. (A, B) DNA-normalized GAG content in DXM- and FA-treated aggregates, respectively. (C, D) DNA-normalized HYP content normalized in DXM- and FA-treated aggregates, respectively. Glucose concentration (g/L) is shown on the y-axis and insulin concentration (µM) on the y-axis. Color indicates the mean normalized value, with the scale shown in each panel.
